# White Tea extract induces lipolytic activity and inhibits adipogenesis in human subcutaneous (pre)-adipocytes

**DOI:** 10.1186/1743-7075-6-20

**Published:** 2009-05-01

**Authors:** Jörn Söhle, Anja Knott, Ursula Holtzmann, Ralf Siegner, Elke Grönniger, Andreas Schepky, Stefan Gallinat, Horst Wenck, Franz Stäb, Marc Winnefeld

**Affiliations:** 1 Research & Development, Research Special Skincare, Beiersdorf AG, Unnastrasse 48, 20245 Hamburg, Germany

## Abstract

**Background:**

The dramatic increase in obesity-related diseases emphasizes the need to elucidate the cellular and molecular mechanisms underlying fat metabolism. To investigate how natural substances influence lipolysis and adipogenesis, we determined the effects of White Tea extract on cultured human subcutaneous preadipocytes and adipocytes.

**Methods:**

For our in vitro studies we used a White Tea extract solution that contained polyphenols and methylxanthines. Utilizing cultured human preadipocytes we investigated White Tea extract solution-induced inhibition of triglyceride incorporation during adipogenesis and possible effects on cell viability. In vitro studies on human adipocytes were performed aiming to elucidate the efficacy of White Tea extract solution to stimulate lipolytic activity. To characterize White Tea extract solution-mediated effects on a molecular level, we analyzed gene expression of essential adipogenesis-related transcription factors by qRT-PCR and determined the expression of the transcription factor ADD1/SREBP-1c on the protein level utilizing immunofluorescence analysis.

**Results:**

Our data show that incubation of preadipocytes with White Tea extract solution significantly decreased triglyceride incorporation during adipogenesis in a dose-dependent manner (n = 10) without affecting cell viability (n = 10). These effects were, at least in part, mediated by EGCG (n = 10, 50 μM). In addition, White Tea extract solution also stimulated lipolytic activity in adipocytes (n = 7). Differentiating preadipocytes cultivated in the presence of 0.5% White Tea extract solution showed a decrease in PPARγ, ADD1/SREBP-1c, C/EBPα and C/EBPδ mRNA levels. Moreover, the expression of the transcription factor ADD1/SREBP-1c was not only decreased on the mRNA but also on the protein level.

**Conclusion:**

White Tea extract is a natural source that effectively inhibits adipogenesis and stimulates lipolysis-activity. Therefore, it can be utilized to modulate different levels of the adipocyte life cycle.

## Background

In the industrialized countries, the rising incidence of obesity-associated disorders including cardiovascular diseases and diabetes constitutes a growing problem. Due to this increase in obesity-related diseases, cellular and molecular processes underlying fat metabolism have been studied extensively in recent years [1,2].

Adipose tissue represents a dynamic endocrine organ that is present throughout the body forming different contiguous or non-contiguous depots [1,3]. It serves as the body's main energy reserve in periods of energy excess and enables fat mobilization during phases of food deprivation. Apart from the regulation of the body's energy balance, factors secreted from adipose tissue play key roles in the modulation of metabolic processes, insulin sensitivity and immunological responses [1].

An increase in adipose tissue mainly involves two processes: an increase in fat cell size (adipocyte hypertrophy) as well as an increase in fat cell number (adipocyte hyperplasia). The formation of mature adipocytes from precursor fat cells designated as preadipocytes is termed the adipocyte life cycle [4-6]. It includes proliferation of preadipocytes, fat cell differentiation (adipogenesis), lipolytic-activity as well as apoptosis of preadipocytes or mature adipocytes.

The complex sequence of preadipocyte differentiation is initially triggered by transcription factor-activated signalling pathways [7]. Three classes of transcription factors are directly involved in adipogenesis: the peroxisome proliferator-activated receptor γ (PPARγ), the CCAAT/enhancer binding proteins (C/EBPα, C/EBPβ and C/EBPδ) and the adipocyte determination and differentiation factor 1 (ADD1/SREBP-1c) [2]. PPARγ is a member of the PPAR subfamily of nuclear hormone receptors. Like all members of the PPAR family, PPARγ functions as an obligate heterodimer with RXR. PPARγ is the most adipose specific of the PPARs and its expression has been shown to be sufficient to induce adipogenesis. In fact, PPARγ is the dominant or 'master' regulator of adipogenesis [7,8]. The second class of transcription factors critically involved in adipocyte differentiation, the C/EBPs, are members of the basic region leucine zipper transcription factor family. Finally, ADD1/SREBP-1c is a member of the basic helix-loop-helix family of transcription factors [9]. In addition to its role in adipogenesis ADD1/SREBP-1c has been associated with the regulation of genes linked to the cholesterol metabolism. In this context, ADD1/SREBP-1c has been termed sterol regulatory element binding protein 1c (SREBP-1c) [10].

In the scientific literature, it is a subject of ongoing debate whether polyphenols and/or xanthines can be utilized to modulate different levels of the adipocyte life cycle [6]. A combination of these different bioactive compounds is naturally present in White Tea extract. In contrast to Green- and Black Tea, White Tea is manufactured only from the buds or first leaves of *Camellia Sinensis *that are plucked and dried with minimal processing. Therefore, the concentrations of epigallocatechin-3-gallate (EGCG) and also methylxanthines (like caffeine) are enriched in White Tea compared to Green- or Black Tea [11]. These ingredients are known to exert biological effects on adipocytes [12-19].

To investigate to what extent this natural plant extract influences adipocyte hypertrophy and adipocyte differentiation, we determined the effects of White Tea extract solution on human preadipocytes and adipocytes. Our data demonstrate the efficacy of this extract on different levels of the cell metabolism that involve modulation of adipogenesis-associated transcription factors.

## Methods

### White Tea extract solution

For our studies, a liquid leaf extract of *Camellia Sinensis *(Actipone^® ^White Tea GW; Lot 11; Symrise, Holzminden, Germany) was used. This extract represents an aqueous solution containing approximately 3% White Tea and comprises high levels of EGCG (0.17%) and several other polyphenols such as epigallocatechin and epicatechin as well as the methylxanthines theobromine and caffeine. For experiments, the extract was diluted in the respective medium (see below) to a final concentration of 0.1%, 0.25%, 0.5%, 0.75% or 2% (v/v). To control for possible glycerol effects, a respective glycerol solution in water was used as control.

### Differentiation of preadipocytes into adipocytes

Subcutaneous human preadipocytes isolated from buttocks, thighs or waists of different healthy subjects were obtained from Cambrex (Verviers, Belgium) or Zenbio Inc. (Research Triangle Park, NC). Cells were cultured according to the manufacturer's instructions. Briefly, cells were incubated in basal growth medium (Cambrex, Verviers, Belgium) containing 10% fetal calf serum, 2 mM L-glutamine, 100 U/ml penicillin and 100 μg/ml streptomycin (Cambrex, Verviers, Belgium) for five (or seven) days at 37°C and 5% (or 7.5%) CO_2_. Cells were seeded into 96-well plates (1 × 10^4 ^per well) or 6-well plates (3 × 10^5 ^per well) and after incubation overnight the differentiation into adipocytes was initiated by addition of 10 μg/ml insulin, 1 μM dexamethasone, 200 μM indomethacin and 500 μM isobutylmethylxanthine (Cambrex, Verviers, Belgium) to the medium. Culture medium containing these ingredients is designated as 'differentiation medium'.

### Determination of cell viability

A viability assay determining the endogenous esterase activity was used to evaluate possible cytotoxic effects of White Tea extract solution and EGCG. Briefly, preadipocytes were cultured for seven days in the presence of 0.1%, 0.25%, 0.5% or 0.75% White Tea extract solution or 50 μM EGCG dissolved in dimethylsulfoxide (DMSO), respectively. Preadipocytes cultured in 'differentiation medium' containing the respective control solution (glycerol or DMSO) served as controls. Subsequently, cells were washed with 1× Dulbecco's Phosphate Buffered Saline (DPBS) (Cambrex, Verviers, Belgium) and incubated for 20 min in 100 μl fluorescein diacetate (FDA) (Sigma, Taufkirchen, Germany) solution (15 μg/ml FDA in 1× DPBS). Fluorescence was then determined at 517 nm in the 96-well plate reader Safire 1 (Tecan, Crailsheim, Germany).

### Determination of triglyceride accumulation

For these experiments, cells were cultivated for seven days in 'differentiation medium' supplemented with 0.1%, 0.25%, 0.5% or 0.75% White Tea extract solution, 50 μM EGCG or the respective control solution.

The accumulation of triglycerides during differentiation was determined on day seven by an AdipoRed Assay (Cambrex, Verviers, Belgium) according to the manufacturer's instructions. Fluorescence was detected at 572 nm and quantified in a 96-well plate reader Safire 1 (Tecan, Crailsheim, Germany).

For additional microscopic analysis, cells were incubated with AdipoRed reagent for 10 min at room temperature. Samples were analyzed by fluorescence microscopy and by phase contrast microscopy using an Olympus IX71 microscope (Hamburg, Germany).

### Determination of glycerol release

Subcutaneous human preadipocytes were cultured according to the manufacturer's instructions as described above. Differentiation to adipocytes was achieved after two weeks of culture in 'differentiation medium'. Prior to incubation with test substances the differentiated cells (1 × 10^4 ^per 96-well) were cultured for one week in Dulbecco's modified Eagle Medium low Glucose (Cambrex, Verviers, Belgium) supplemented with 1% Bovine Albumin Fraction V, 100 U/ml penicillin, 100 μg/ml streptomycin and 1× Glutamax (all obtained from Gibco/BRL, Eggenstein, Germany). Culture medium containing these ingredients is designated as 'maintenance medium'.

For experiments, cells were incubated in 150 μl 'maintenance medium' containing either 2% White Tea extract solution or control solution for 24 hrs at 37°C and 7.5% CO_2_. The medium was removed and cells were washed twice with phosphate buffered saline (PBS). Cells were incubated for another five days in 'maintenance medium' prior to quantification of glycerol release. For every donor, seven samples were prepared both for control and White Tea incubation.

Free glycerol reagent and standard solution (Sigma, Taufkirchen, Germany, standard dilution: 125 to 1.95 μg/ml (1:1 steps)) were used according to the manufacturer's instructions. For measurement either 120 μl supernatant or standard solution were used for each sample and mixed with 100 μl of free glycerol reagent in a 96-well. After 15 min incubation at room temperature in the dark, absorption was measured in a 96-well plate reader Spectra MAX Plus (Molecular Devices, Union City, California, USA) at 540 nm.

### Gene expression of adipogenesis-associated transcription factors

Subcutaneous human preadipocytes were cultured as described above. For experiments, cells were cultivated for five days in 'differentiation medium' with or without 0.5% White Tea extract solution. Cells were harvested on day five after induction of differentiation and homogenized in TRIzol^® ^(Invitrogen, Karlsruhe, Germany) following the manufacturer's protocol. After reverse transcription, samples were analyzed for the following transcription factors PPARγ, ADD1/SREBP-1c, C/EBPα, C/EBPβ and C/EBPδ by Real-Time TaqMan^®^-PCR using the 7900 HT Fast-Real-Time PCR System (Applied Biosystems, Darmstadt, Germany).

FAM labelled primers for the qRT-PCR (Applied Biosystems, Forster City, California, USA) were as follows: Inventoried TaqMan Assays for the internal control glyceraldehyde-3-phosphate dehydrogenase (GAPDH; Hs99999905_m1), for the target RNA PPARγ (Hs00234592_m1), ADD1/SREBP-1c (Hs01088691_m1), C/EBPα (Hs00269972_s1), C/EBPβ (Hs00270923_s1) and C/EBPδ (Hs00270931_s1). PCR conditions were as follows: 50°C for 2 min, 94.5°C for 10 min followed by 40 cycles at 97°C for 30 sec and 59.7°C for 1 min. Real-time PCR data were analyzed using the Sequence detector version 2.3 software supplied with the 7900 HT Fast-Real-Time PCR System (Applied Biosystems, Darmstadt, Germany). Quantification was achieved using the 2^-ΔΔCt ^method which calculates the relative changes in gene expression of the target normalized to an endogenous reference (GAPDH) and relative to a calibrator that serves as the control group.

### Immunofluorescence microscopic analysis

For immunofluorescence analysis, preadipocyte populations were incubated in 'differentiation medium' with or without 0.5% White Tea extract solution for 10 days as described above. Cells were grown on coverslips and for Incell-Western analysis in 96-well plates. In the following, cells were fixed with 4% formaldehyde solution for 30 min at room temperature, washed with PBS and permeabilized with 0.2% Triton X-100. After successive washing with PBS, fixed cells were pre-treated with PBS containing 10% donkey-serum for 30 min. Cells were then incubated for 1 h with primary antibodies directed against ADD1/SREBP-1c (sc 8984; Santa Cruz, Heidelberg, Germany) and GAPDH (sc-47724; Santa Cruz, Heidelberg, Germany). Coverslips and 96-well plates were successively rinsed three times with PBS, and then incubated for 1 h with secondary antibodies labelled with Cy3 (microscopic analysis), IRDye680 or IRDye-800 (Incell-Western analysis). Results were determined using the Fluorescence microscope IX71 in combination with the software cell^F v. 2.4 (Olympus, Hamburg, Germany). Incell-Western analyses were carried out using the Odyssey Infrared Imager (Li-Cor Biosciences, Bad Homburg, Germany).

### Determination of Sirt1 gene expression

Human preadipocytes were cultured in 'differentiation medium' in the absence or presence of 0.5% White Tea extract solution as described above. Cells were harvested on day five after induction of differentiation and homogenized in TRIzol^® ^(Invitrogen, Karlsruhe, Germany) following the manufacturer's protocol. After reverse transcription, samples were analyzed for Sirt1 by Real-Time TaqMan^®^-PCR using the 7900 HT Fast-Real-Time PCR System (Applied Biosystems, Darmstadt, Germany).

FAM labelled primers for the qRT-PCR (Applied Biosystems, Forster City, CA) were as follows: Inventoried TaqMan Assays for the internal control GAPDH (Hs99999905_m1) and for the target RNA Sirt1 (Hs01009006_m1). PCR conditions were as follows: 50°C for 2 min, 94.5°C for 10 min followed by 40 cycles at 97°C for 30 sec and 59.7°C for 1 min. Real-time PCR data were analyzed using the Sequence detector version 2.3 software supplied with the 7900 HT Fast-Real-Time PCR System (Applied Biosystems, Darmstadt, Germany). Quantification was achieved using the 2^-ΔΔCt ^method which calculates the relative changes in gene expression of the target normalized to an endogenous reference (GAPDH) and relative to a calibrator that serves as the control group.

### Statistical analysis

A significance level of 0.05 (alpha) was chosen for statistical analysis, based on two-sided hypothesis testing. For the analysis SAS software package for Windows V9.1.3 was used.

Depending on the parameter investigated the following analyses were conducted:

#### Determination of triglyceride accumulation, ADD1/SREBP-1c protein expression and cell viability

• Test of normality using Shapiro-Wilk's test

• If rejection of the normality hypothesis: analysis of the Blom-transformed ranks of the original data, otherwise analysis of original data

• Comparison versus control via repeated measures analysis of variance with qualitative factor treatment

#### Determination of glycerol release

• Comparison versus control by means of Wilcoxon signed rank test

## Results

### White Tea extract solution decreases triglyceride accumulation without affecting cell viability

**Figure 1:**
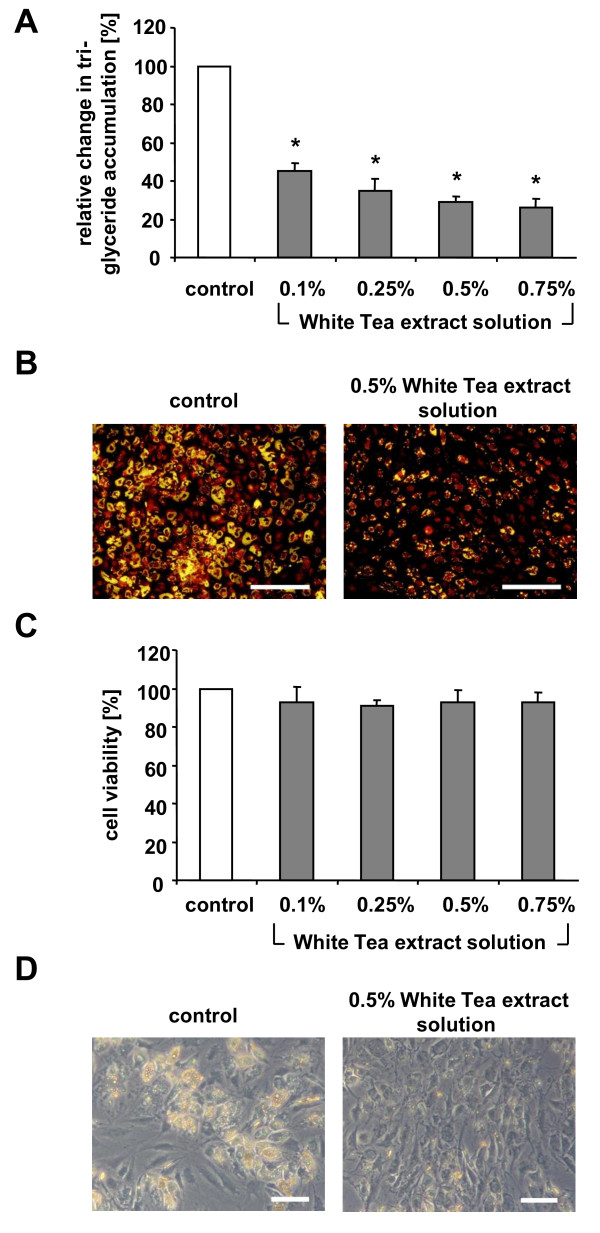
**Effects of White Tea extract solution on triglyceride accumulation during preadipocyte/adipocyte differentiation**. (A) Triglyceride accumulation and (C) cell viability of maturing preadipocytes incubated with different amounts of White Tea extract solutions (0.1%, 0.25%, 0.5% and 0.75%) is shown relative to untreated control cells set as 100%. For experiments, ten independent cell cultures were prepared both for control and White Tea extract solution incubation (n = 10). Results are depicted as mean ± SD. Significant differences are marked with an asterisk (* for p < 0.0001). (B) Images after triglyceride staining (yellow) from cell populations incubated with and without 0.5% White Tea extract solution. Scale bar: 200 μm. (D) Displayed are phase contrast images from cell populations cultivated in the absence or presence of 0.5% White Tea extract solution. Scale bar: 50 μm. For our studies we used cells up to the third passage.

To investigate effects of White Tea extract on triglyceride accumulation during human preadipocyte/adipocyte differentiation in vitro, cells were cultured in 'differentiation medium' for seven days (n = 10) in the absence or presence of 0.1%, 0.25%, 0.5% or 0.75% White Tea extract solution. The total amount of lipid accumulation was determined (Figure 1A). Cells treated with White Tea extract solution dose-dependently exhibited significantly decreased triglyceride levels (p < 0.0001). Compared to control cells (set as 100%), White Tea extract solution reduced triglyceride accumulation up to 70%.

As illustrated in Figure 1B, the majority of control cells accumulated triglycerides (yellow staining) in the lipid droplets. These cells are characterized by lipid droplets filling almost the entire cell while the cytoplasm is being shifted to the periphery. In contrast, cells incubated with 0.5% White Tea extract solution did not accumulate lipid droplets as indicated by the absence of yellow staining. The few vesicles that could be detected were smaller in size compared to the respective control.

In addition, a viability assay was used to determine any possible adverse effects of White Tea extract solution (n = 10). Control cells (set as 100%) and preadipocytes cultivated in 'differentiation medium' with 0.1%, 0.25%, 0.5% or 0.75% White Tea extract solution displayed a comparable esterase activity indicating that the viability of cultured cells was not affected by incubation with White Tea extract solution (Figure 1C). These results are in good agreement with observations from phase contrast microscopy shown in Figure 1D. Cell populations cultivated in 'differentiation medium' in the presence (0.5%) or absence of White Tea extract solution grew to comparable cell densities.

### White Tea extract solution increases glycerol release

**Figure 2:**
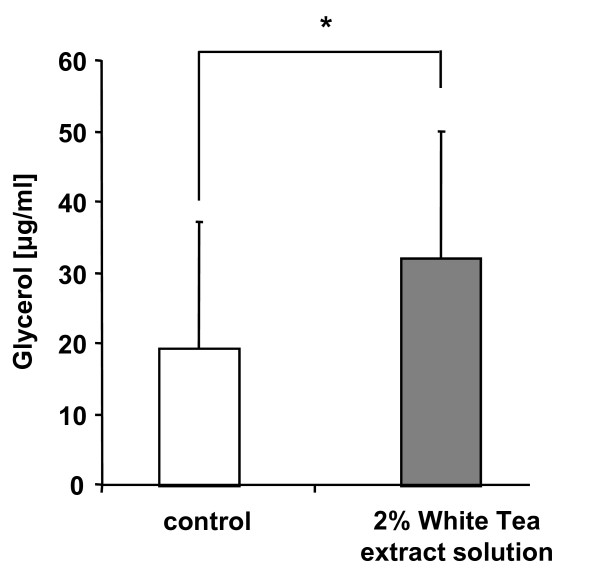
**Determination of glycerol release in differentiated adipocytes after incubation with White Tea extract solution**. Glycerol content is given in μg/ml. For experiments, seven independent cell cultures were prepared both for control and White Tea extract solution incubation (n = 7). Results are depicted as mean ± SD. Significant differences are marked with an asterisk (* for p ≤ 0.05). For our studies we used cells up to the third passage.

To address the question whether White Tea extract solution also stimulates lipolysis-activity in differentiated adipocytes, we determined if incubation of these cells (n = 7) with White Tea extract solution influences the degradation of triglycerides. As our studies show, treatment of adipocytes with 2% White Tea extract solution significantly (p = 0.016) increased the content of free glycerol to 32 (± 18) μg/ml as compared to control cells at 20 (± 18) μg/ml (Figure 2).

### Effect of White Tea extract solution on ADD1/SREBP-1c protein expression

**Figure 3:**
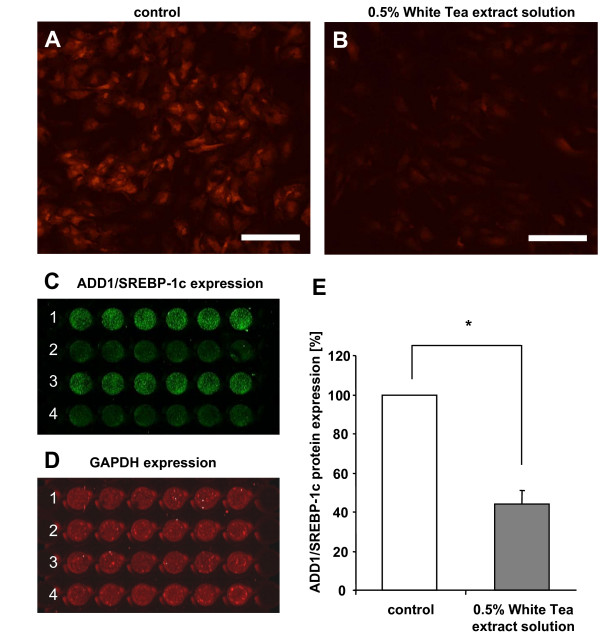
**Effect of White Tea extract solution on ADD1/SREBP-1c expression during adipogenesis**. Preadipocyte populations (0 d) were incubated with differentiation medium without (A) and with (B) 0.5% White Tea extract solution for 10 days. A and B: ADD1/SREBP-1c was detected by immunofluorescence microscopic analysis. Scale bar: 200 μm. C-E: Quantitative analyses were performed using the Odyssey Infrared Imager. 1 and 3: Cells incubated in control solution. 2 and 4: Cells incubated in the presence of White Tea extract solution. For experiments, 17 independent cell cultures were prepared both for control and White Tea extract solution incubation (n = 17). Results are depicted as mean ± SD. Significant differences are marked with an asterisk (* for p < 0.0001).

Since our results (Figure 1A–D) indicated a White Tea-mediated reduction of triglyceride accumulation during adipogenesis we investigated the underlying mechanism of action in more detail. Therefore, we determined the protein level of an essential adipogenic transcription factor – ADD1/SREBP-1c – by immunofluorescence analysis. Interestingly, stimulation with White Tea extract solution substantially down-regulated ADD1/SREBP-1c protein expression during adipogenesis compared to control cells (Figure 3A and 3B). Moreover, Incell-Western analyses were performed to quantify ADD1/SREBP-1c expression. Compared to controls (set as 100%), cells incubated in the presence of 0.5% White Tea extract solution showed a significantly (p < 0.0001) decreased ADD1/SREBP-1c expression to 44 (± 6)% (Figure 3C, E) whereas GAPDH expression was not affected (Figure 3D).

### Effects of White Tea extract solution on the gene expression of adipogenesis-associated transcription factors

**Figure 4:**
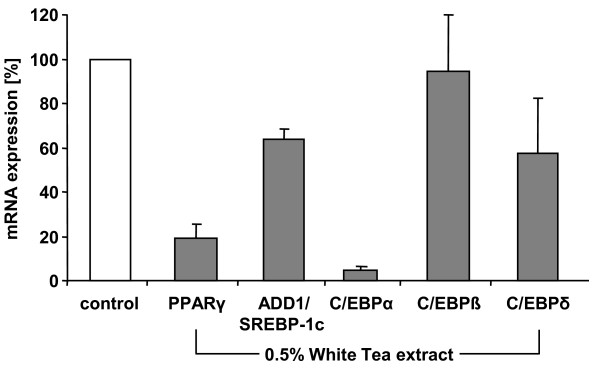
**Effects of White Tea extract solution on gene expression of adipogenesis-associated transcription factors**. Gene expression of PPARγ, ADD1/SREBP-1c, C/EBPα, C/EBPβ and C/EBPδ in differentiating preadipocytes after incubation with White Tea extract solution compared to control cells set as 100%. Expression of each gene is normalized to GAPDH. Three independent experiments were prepared both for control and White Tea extract solution incubation (n = 3). Results are depicted as mean ± SD. For our studies we used cells up to the third passage.

To gain insight into the molecular events associated with the above described inhibition of adipocyte differentiation, cells were cultivated in the presence or absence of 0.5% White Tea extract solution for five days after initiation of differentiation and gene expression of the transcription factors PPARγ, ADD1/SREBP-1c, C/EBPα, C/EBPβ and C/EBPδ was determined relative to control cells by quantitative RT-PCR (Figure 4; n = 3). Compared to control cells (set as 100%), cells incubated with White Tea extract solution display a decrease in mRNA levels of PPARγ (19% ± 6%), ADD1/SREBP-1c (64% ± 5%), C/EBPα (5% ± 2%) and C/EBPδ (58% ± 25%). The expression of C/EBPβ mRNA, however, was not affected (95% ± 26%).

### White Tea extract solution decreases Sirt1 gene expression

**Figure 5:**
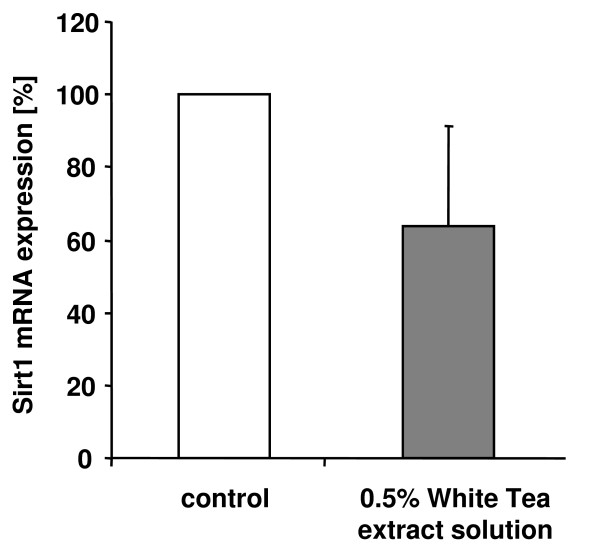
**White Tea extract solution decreases Sirt1 gene expression**. Sirt1 gene expression in differentiating preadipocytes after incubation with 0.5% White Tea extract solution is shown relative to untreated control cells set as 100%. Gene expression is normalized to GAPDH. Three independent experiments were prepared both for control and White Tea extract solution incubation (n = 3). Results are depicted as mean ± SD. For our studies we used cells up to the third passage.

The enzyme Sirt1 has been implicated in the process of adipogenesis [20]. Accordingly, we investigated whether the effects of White Tea extract solution on adipogenesis are paralleled by an altered Sirt1 gene expression. Compared to the untreated controls (set as 100%), cells incubated with 0.5% White Tea extract solution (n = 3) displayed a decrease in Sirt1 mRNA levels to 64 (± 28)% (Figure 5).

### Effects of EGCG on triglyceride accumulation and cell viability

**Figure 6:**
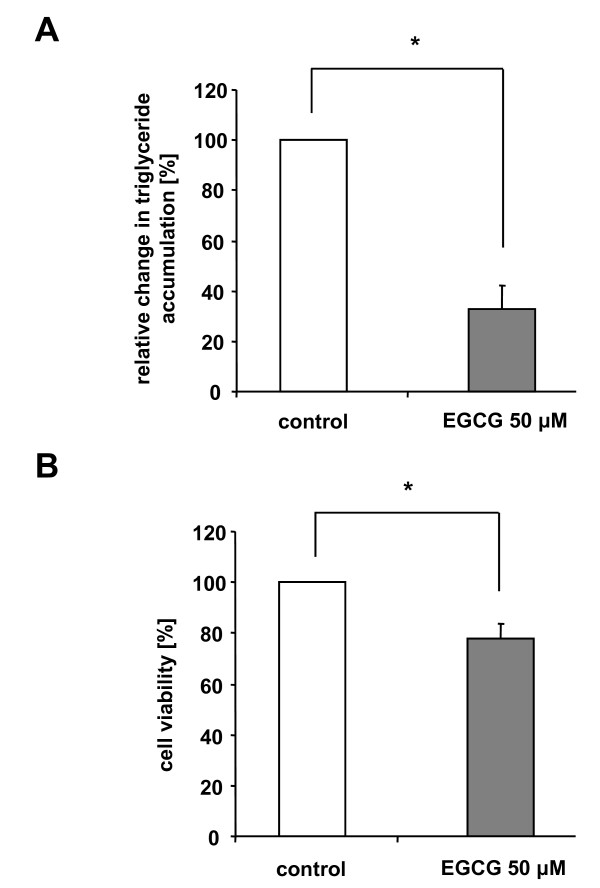
**Decrease in triglyceride concentration and cell viability in differentiating preadipocytes after stimulation with EGCG**. (A) Triglyceride content in differentiating preadipocytes after stimulation with 50 μM EGCG is shown relative to untreated control cells set as 100%. For experiments, ten independent cell cultures were prepared both for control and EGCG incubation (n = 10). Results are depicted as mean ± SD. Significant differences are marked with an asterisk (* for p < 0.0001). (B) Decreased viability in differentiating preadipocytes after stimulation with 50 μM EGCG compared to control cells. Cell viability is shown relative to untreated control cells set as 100%. For experiments, ten independent cell cultures were prepared both for control and EGCG incubation (n = 10). Results are depicted as mean ± SD. Significant differences are marked with an asterisk (* for p < 0.0001). For our studies we used cells up to the third passage.

The White Tea extract solution used in our studies contained as a main component EGCG. In order to investigate the possibility that EGCG is responsible for the reduced adipocyte differentiation observed in cell populations cultivated in the presence of White Tea extract solution, we analyzed EGCG effects. As shown in Figure 6A, cells cultured for seven days (n = 10) in 'differentiation medium' containing 50 μM EGCG displayed reduced triglyceride levels (p < 0.0001) of 33 (± 10)% compared to control cells (set as 100%).

In contrast to cell populations incubated with White Tea extract solution, maturing preadipocytes (n = 10) cultured in the presence of 50 μM EGCG showed an esterase activity of 78 (± 6)% compared to control cells (set as 100%) indicating that EGCG slightly impairs cell viability during adipogenesis (Figure 6B)(p < 0.0001).

## Discussion

In the human body, adipose tissue is present as both visceral and subcutaneous fat. Apart from other functions, its main role is associated with the storage of energy. In periods of food overabundance, human adult adipose tissue maintains its ability to increase the number of adipocytes suggesting that differentiation of preadipocytes can occur at any time in response to nutritional or hormonal stimulants [3].

A large body of evidence indicates that certain plant extracts and their respective bioactive components might have direct effects on adipose tissue [6]. Based on these data, the overall objective of our studies was to investigate the effects of a particular White Tea extract solution on fat accumulation and the expression of adipocyte-specific genes in primary human preadipocyte and adipocyte culture.

In previous studies polyphenols as well as various natural extracts were shown to induce apoptosis, decrease lipid accumulation and stimulate lipolysis in preadipocytes and adipocytes [6,13,17]. These results, however, were mainly obtained studying 3T3-L1 cells. Although this mouse cell line represents a well-established model system to study fat metabolism [21], 3T3-L1 cells differ in various aspects from primary cells isolated from fat tissue. Furthermore, it has been widely accepted that significant differences in adipose tissue biology exist between species. To account for these known differences, we exclusively utilized primary cultured human cells for our experiments. Compared to murine 3T3-L1 cells, human preadipocytes and adipocytes should better reflect the human lipid metabolism and, hence, the human in vivo situation.

Natural White Tea represents the least processed form of tea and comprises high levels of EGCG and several other polyphenols such as epigallocatechin and epicatechin as well as the methylxanthines theobromine and caffeine.

As our data show, incubation with White Tea extract solution decreases triglyceride incorporation in human preadipocytes during adipogenesis (Figure 1A, B). Importantly, this significant reduction in triglyceride accumulation is not due to toxic side effects (Figure 1C, D).

One possible explanation for the observed attenuation in triglyceride incorporation during adipogenesis could be the ability of White Tea extract solution to stimulate lipolytic activity as evidenced by an increased conversion of triglyceride to fatty acids and glycerol. Interestingly indeed, by measuring glycerol release we were able to demonstrate that White Tea extract solution augments lipolysis-activity in differentiated adipocytes (Figure 2). However, the degree of lipolysis-activity induced by White Tea extract solution is not sufficient enough to fully explain the observed reduction in triglyceride incorporation. In addition to this effect one could hypothesize that White Tea extract solution also inhibits adipogenesis on a molecular level. Accordingly, we investigated the effects of White Tea extract solution on the expression pattern of ADD1/SREBP-1c an essential adipogenesis-related transcription factor. ADD1/SREBP-1c is highly expressed in the liver and in adipose tissue and plays an important role in adipocyte differentiation [9]. ADD1/SREBP-1c mRNA levels are increased in determined preadipocytes and to a greater extend augmented during the differentiation process [22]. Interestingly, ADD1/SREBP-1c has been shown to promote PPARγ expression [1,2] further stressing its physiological relevance.

To study ADD1/SREBP-1c regulation in more detail, we investigated its protein expression following treatment with White Tea extract solution. Compared to differentiated adipocytes, that display an augmented ADD1/SREBP-1c expression (control, Figure 3A), cells incubated in the presence of White Tea extract solution show only a weak ADD1/SREBP-1c signal (Figure 3B). This observation together with quantitative analysis (Figure 3C–E) clearly shows that White Tea extract solution influences adipocyte differentiation.

Beside ADD1/SREBP-1c, several transcription factors are involved in orchestrating adipocyte differentiation [2,3]. Briefly, adipogenesis is initiated by the transient expression of C/EBPβ and C/EBPδ. In response to adipogenic signals, these transcription factors lead to the activation of PPARγ which in turn stimulates the expression of C/EBPα. C/EBPα again exerts positive feedback on PPARγ to maintain the differentiation process [8].

To gain more mechanistic insights into the signalling cascade that is stimulated by White Tea extract solution, we investigated the expression of the essential transcription factors PPARγ, ADD1/SREBP-1c, C/EBPα, C/EBPβ and C/EBPδ. Interestingly, our results illustrate that cells incubated with White Tea extract solution display a decrease in PPARγ, ADD1/SREBP-1c, C/EBPα and C/EBPδ mRNA levels during adipogenesis while the expression of C/EBPβ mRNA was not affected (Figure 4). Overall, the observed reduction of adipogenesis-related transcription factors supports the notion that White Tea extract solution acts on two different levels, by increasing lipolysis and by inhibiting adipogenesis.

However, it should be noted that the results presented here were achieved using subcutaneous human (pre)-adipocytes. When using visceral human (pre)-adipocytes, neither White Tea extract solution nor EGCG significantly reduced triglyceride accumulation (data not shown). One explanation for this phenomenon could be that PPARγ activity is considerably lower in primary human visceral adipocytes [23] compared to subcutaneous adipocytes. Since our results show that White Tea extract solution acts by decreasing PPARγ mRNA expression in subcutaneous (pre)-adipocytes these data might explain the different actions observed in subcutaneous and visceral cells.

Another interesting facet that is a matter of intense research is the role of sirtuins in fat metabolism. Sirtuins belong to a family of enzymes implicated in apoptosis and fatty acid metabolism, to name two physiological functions. Mammalian sirtuins comprises of the seven members, Sirt1 – Sirt7 [24]. Sirt1 regulates adipogenesis by inhibiting the expression of genes that control adipocyte differentiation and also triglyceride accumulation in 3T3-L1 cells. Sirt1 over-expression results in a reduction of PPARγ, C/EBPα and C/EBPδ but not C/EBPβ mRNA indicating that Sirt1 functions as a repressor of genes that control adipocyte differentiation [20].

Along these lines it is interesting to note that the established Sirt1 activator resveratrol, a polyphenol, inhibits adipogenesis in maturing 3T3-L1 preadipocytes [25]. Accordingly, it appears plausible that polyphenols present in White Tea extract solution could reduce adipogenesis by means of an increased Sirt1 enzyme expression.

To tackle this question we determined Sirt1 mRNA expression after stimulation with White Tea extract solution. Interestingly, our data showed a slight reduction in Sirt1 gene expression suggesting that the polyphenols present in White Tea extract solution do not reduce adipogenesis via modulation of Sirt1 levels (Figure 5). The observed decrease in Sirt1 mRNA levels could be explained by the fact that Sirt1 expression is per se increased during fat cell differentiation [20]. Therefore, a decrease in adipogenesis induced by White Tea extract solution could indirectly affect Sirt1 mRNA expression.

Moreover, no increase in Sirt1-activity was detected in the presence of 0.5% White Tea extract solution using a cell free Sirt1 activity assay (unpublished data). However, further studies need to be conducted in the future to elucidate the exact mechanism in more detail and to corroborate this finding.

Our results discussed above show that treatment of primary human (pre)-adipocytes with White Tea extract solution modulates both adipogenesis and lipolysis. These White Tea extract solution-induced effects are associated with a decrease in the expression of adipogenesis-associated transcription factors as well as a diminished gene expression of Sirt1. One possible explanation to account for these rather complex effects could be the fact that White Tea extract solution is composed of several active ingredients, among them polyphenols and methylxanthines.

To elucidate the possible effects of polyphenols in more detail, we investigated the influence of EGCG on triglyceride accumulation during differentiation in human pre-adipoyctes. EGCG represents the most abundant catechin present in White Tea extract solution. In that context it is important to remark that conflicting data have been published with respect to the effects of EGCG on (pre-)adipocytes. While EGCG inhibits adipogenesis in 3T3-L1 cells [15] it did not affect the conversion of preadipocytes to adipocytes in human AML-1 cells [16]. Our results using human primary preadipocytes show that EGCG reduces triglyceride incorporation during adipogenesis (Figure 6). Considering our hypothesis the EGCG-mediated decrease in triglyceride accumulation might be due to an EGCG-induced inhibition of adipogenesis.

Previous studies also showed that EGCG did not promote lipolysis in both 3T3-L1 [13] and C3H10T1/2 cells [14]. These observations indicate that the stimulated lipolytic activity induced by White Tea extract solution might not be mediated by EGCG.

On the other hand, methylxanthines have been reported to stimulate triglyceride conversion by inhibiting phosphodiesterase 3B activity and subsequently increasing cAMP levels [18,19]. Unpublished data from our lab show that caffeine (5 mM) increases lipolysis-activity in human adipocytes. Therefore, the observed White Tea-mediated increase in lipolysis-activity might, at least in part, be caused by methylxanthines.

Adding more to the complexity, EGCG not only influences triglyceride accumulation during adipogenesis but also the process of apoptosis. In both 3T3-L1 and AML-1 cells stimulation with EGCG induced apoptotic cell death [15,16]. Our observation that the viability of primary human preadipocytes is reduced in the presence of EGCG appears to be in line with these studies. However, treatment with White Tea extract solution did not impair cell viability. Although we did not specifically study apoptosis, it can be speculated that other natural substances present in White Tea extract solution might counteract the EGCG-mediated reduction of cell viability.

## Conclusion

The increase of obesity-related diseases highlights the need to further investigate the cellular and molecular mechanisms underlying fat metabolism. In this context, we determined the effects of a particular White Tea extract solution on fat accumulation and the expression of adipocyte-specific genes in primary human preadipocyte and adipocyte cultures.

Overall, our data demonstrate that White Tea extract solution effectively inhibits adipogenesis and stimulates lipolysis-activity. This plant extract is, therefore, an ideal natural source to modulate the adipocyte life cycle at different stages and to induce anti-obesity effects.

## Competing interests

The authors declare that they have no competing interests.

## Authors' contributions

JS performed the experiments concerning adipogenic transcription factors. Moreover, he assisted with interpretation of the results and helped draft the manuscript. AK analyzed the lipolytic activity of differentiated adipocytes. RS and UH analyzed the triglyceride accumulation during adipocyte differentiation. EG performed (in cooperation with JS) LDA-analysis (adipogenic transcription factors). AS, SG, HW, FS assisted with interpretation of the results. MW supervised the analyses and helped to draft the manuscript. All authors read and approved the final manuscript.
